# Review and Abstracts from a Collection of Memoirs upon an Important Discovery of Some of the Unknown Functions of the Pancreas

**Published:** 1865-03

**Authors:** Lucien Corvisart

**Affiliations:** Physician to the Emperor, Officer of the Legion of Honor, &c., &c.


### REVIEW AND ABSTRACTS FROM A COLLECTION OF MEMOIRS UPON AN IMPORTANT DISCOVERY OF SOME OF THE UNKNOWN FUNCTIONS OF THE PANCREAS.

By Professor LUCIEN CORVISART, Physician to the Emperor, Officer of the Legion of Honor, &c., &c.

Translated for the St. Louis Medical and Surgical Journal, By J. K. BAUDUY, M.D., St. Louis, Mo.

A recent publication by Victor Mason et Fils, of Paris, has greatly interested us. Lucien Corvisart, in contributing to medical science his collection of memoirs, upon a function so obscure as that of the pancreas, has greatly added to our previous knowledge upon the subject, and has thrown many additional rays of light upon a study which has so long been veiled in profound darkness, and hidden from the most penetrating and indefatigable searches of physiological exploration. This little book will not fail to interest the profession at large, and will certainly greatly repay them for the attention they may bestow upon it. The more arduous the avenues of approach to certain medical subjects, the more intricate and unintelligible their comprehension, the greater are the energy and perseverence which have always been displayed by our profession, in overcoming all the obstacles and difficulties, in the way of clear elucidations of obtruse and doubtful problems. The pancreatic function has long been a source of uncertainty and dispute among physiologists, and the task of clearing away many of the clouds that have so obstinately hung around this subject replete with so much interest, and *pathological importance*, was reserved for the genius of Corvisart. He does not arrogate to himself, however, the claim of the first discovery of the functions to be described, as the idea first originated with Purkinje and Pappenheim, in 1836, but he only has established and *proved* laws which govern the pancreatic function, that are based upon experiments which were probably suggested by his familiarity with their views. At all events, great credit is due to him for his scientific and successful elaboration of views, be the original conceiver of them whomsoever he may. Nor does the author undertake to explain the entire physiology of pancreatic functions, but merely professes to have ascertained some very important and interesting uses of the gland, based upon numerous proofs furnished by his investigations, and resting upon the broad basis of scientific experiments, which all who may feel inclined to be incredulous, may repeat for themselves in their own studios. Our previous knowledge of this organ, the author does not entirely controvert—he contents himself with boldly maintaining *new truths*, which, as zealous devotees in pursuit of professional knowledge, we ought carefully to collect, weigh, and balance, before we finally either receive or reject. The weight of authority in France and Germany, tends to strengthen and confirm the position taken by the author. The first statement that attracts our attention, and causes us to pause and reflect in reading the memoirs, is the close *analogy* said to exist between pancreatic and gastric digestion. The author then goes on to say, that the most important function of the pancreas is the digestion of *nitrogenized elements of food.* These constitute the principal topics discussed in his little work, though there are, however, some secondary but very useful points which are taken into consideration, the principal of which are as follows, viz.:—

"1st. The *intra venous digestion of the blood itself.*"

This form of digestion resulting, perhaps, in the constant renovation of this fluid by means as singular as they are little understood.

"2d. The fact that certain azotized alimentary substances are *digested in variable quantities*, this depending as to whether the pancreas or stomach is in the better state of preservation, and which of the two organs most completely retains and performs its digestive functions.

"3d. The influence of stomachic digestion (that is to say, the normal formation by it of gastric peptones) upon *the production of the pancreatic ferment*, the quantity and quality of which are of so much importance to the welfare of the economy.

"4th. A secondary, but very interesting point, is that of pyloric *insufficiency*, as a cause of either gastric or intestinal dyspepsia."

Owing to my small amount of space, it will be impossible to give a full description of the author's opinions. I shall therefore be obliged to confine myself to the principal topics of interest and importance he discusses. I shall often quote his own words, in order to be accurate and in exact conformity with his precise meaning, and I will be likewise compelled to give very literal translations to avoid all possibility of misrepresenting his views.

The subject is so new, and so little able are the generality of the profession to pronounce *definitely* at present upon the topics under discussion, that it will be useless to offer comments and criticisms upon *experimental deductions* which we cannot fully appreciate until we convince ourselves by the same course of investigations pursued by the author, which is open to all inquiring minds. Our previous knowledge of the pancreatic function consisted in what we were taught to believe was its action on amylaceous matters, converting them into dextrin and glucose, with its additional and perhaps more important office, that of emulsifying *fatty matters.*

As I have already stated, the first discoverers of the influence exerted by pancreatic digestion upon azotized aliments, were admitted by the author to be Purkinje and Pappenheim, in 1836, whose views were condemned afterwards, however, by all physiologists, and finally were somewhat modified and confused by Claude Bernard, in consequence of two mistakes made whilst experimenting with the results of putrefaction. Great are the advantages to be derived from the perusal of this learned work of Corvisart, whose laborious researches and five years of indefatigable study, at least entitle it to our attentive consideration. In addition to all this, its weight and authority are unquestionable, when we consider the high professional attainments, and elevated position so long and usefully occupied by the distinguished author. Nor are the advantages to be gained by us in weighing these subjects merely imaginary, but they are *practical*, because the doctrines inculculated are not useless theoretical expositions; on the contrary, the author has drawn *pathological deductions* from his experiments which must interest all practitioners of medicine, and obviate many of the difficulties encountered by them in the diagnosis and treatment of the various causes of dyspepsia.

In the words of the author, "in the present state of our knowledge, what precise information have we to guide us concerning intestinal dyspepsia, its causes, mechanism, and rational treatment?" Corvisart's views, which at first met with obstinate incredulity and rigid criticisms, are every day gaining converts abroad, and many eminent physiologists, such as Meissner, Wittish, and Bach, Danilewski, Stokwis in Germany; Harley in England; Prof. Schiff in Switzerland, &c., have fully confirmed, by repeated experiments the views of the author. Longet has not only adopted and sanctioned Corvisart's contribution to our knowledge of the physiology of digestion, but he publicly teaches them to his large classes. To return to the author's opinions again, I shall continue by freely quoting his own words upon the principal topics with which it behooves us to become acquainted. The pancreas is, as it were, a supplementary organ, whose action after a large repast is auxiliary of gastric digestion. Pancreatic digestion, however, is independent of gastric digestion. The former does not merely consist in transforming into other peptones, those which have already been acted upon in the stomach. In other words, gastric peptones do not undergo changes in consequence of the pancreatic juice, but on the contrary, the author positively maintains, that when a nitrogenized element of food, or a portion of one, has been completely submitted to the process of gastric digestion, it is almost invariably, *directly* absorbed by the stomach. "There are gastric peptones and pancreatic peptones." Corvisart entirely rejects the idea entertained by certain physiologists, who assert that the stomach only macerates, or subdivides the food without dissolving it; he maintaining, on the contrary, that every article of albumenoid nourishment submitted to the action of the stomach, is not divided, but *dissolved*, passes through a filter, as it were, and is absorbed by the membranes themselves. In fact, he adds that the pancreatic function comes into play upon portions of albumenoid substances which have left the stomach *before* any transformation had taken place by gastric influence, and that in certain cases the action of the pancreas is so considerable as fully to equal that of the stomach. Here, the author remarks, that as regards the mere quantity of secreted digestive fluids, care should be taken to avoid fallacious conclusions therefrom, because the stomach is apt to be regarded as much more powerful, from the fact of the gastric secretion being sometimes more *abundant* than the pancreatic fluid; but by way of compensation, he observes that the pancreatic fluid is *ten times* stronger and higher in its possession of ferment (pancreatine). The pancreatic fluid enjoys the remarkable privilege of acting much more *rapidly* upon azotized aliments, whether they be alkaline, neutral, or acid. It is strange that these functions are so entirely independent of each other, that the pancreatic fluid alone is able to accomplish the digestion of food which has not been subjected to gastric influences. Hence, albumenoid matters, without any preparation whatsoever, directly placed in the intestine in a crude state, or in the pancreatic juice outside of the body, will, in both places, be perfectly and completely digested. Moreover, to acquire this digestive property, the pancreatic juice has not to be mixed with either the "succus intestinalis," or the bile. The author clearly proves that this process may be accomplished outside of the body with the pancreatic secretion alone, or isolated pancreatine, and that the function will be performed as certainly as it would be were it exercised in the interior of the duodenum itself. As long as the gastric and pancreatic juices are separated, and act successively, they exercise their respective functions perfectly, and the quantity of albuminose resulting may be almost doubled; but it is a remarkable fact, that if in a *pure* state the two digestive ferments simultaneously meet, the respective digestions will cease to progress so freely. Far from the digestive products being increased or doubled by the circumstances of the mingling of these juices, they will, on the contrary, be greatly diminished; because, in this state, which is not a physiological one, the pepsine and pancreatine will neutralize one another. It may here be objected, that this union of the juices can not well be prevented, but Corvisart proves that nature guards against this contingency by three different methods, viz.:—

1st. By means of the pylorous which separates the two ferments.

2d. By gastric digestion itself, by means of which the pepsine in forming peptone becomes exhausted and consumed.

3d. By the bile, in which, as Pappenheim has demonstrated, destroys the activity of the gastric ferment.

By this last assertion, the author does not mean that the bile precipitates the mass of peptones, produced by the action of the stomach, so that digestion is destroyed, and has to re-commence; but, on the contrary, *the bile itself* is precipitated, by the acidity of the gastric juice or chyme; finally, very little gastric peptones arrive in the intestine, as the stomach has previously absorbed them.

*Intra-venous digestion* is so beautifully and satisfactorily explained, that I will quote the author's own words, verbatim:—

"During *the first three hours* that follow a meal, a period during which digestive *dissolution*, transformation, and absorption, are not far advanced, the blood of the portal vein (compared with the venous circulation at large) does not become enriched with a sensible quantity of nitrogenized materials by digestive absorption; on the contrary, in the intestine under the influence of the alkaline pancreatic juice, the elements of the blood, fibrine, and globules, become transformed into albumen, (caseiforme) by a commencement of digestion.

"But if it be taken into consideration that during the first three hours of digestion: Firstly, that the pancreatic juice poured out into the duodenum remains there in a pure and active condition; Secondly, that it is able to pass into the portal vein, because absorption by the mesenteric veins is not suspended; Thirdly, that the pancreatic secretion can exercise its digestive action on an alkaline medium as that of blood. If one takes into consideration, on the other hand, that it is precisely during these first three hours of digestion that a great part of the globules and fibrine of the blood of the portal vein are formed *in equal proportions* inside of this vein into albumen, (commencement of a similar transformation to that which they underwent in the intestine, under the influence of the same pancreatic juice), it is difficult to recoil before the hypothesis of a *veritable intra-venous digestion*, a function which is new to us, and which it is necessary to study. No real difference has been traced between the nitrogenized matters called extractives, and the albuminose produced by gastric or pancreatic digestion. The chyliferous vessels, *the portal vein and its continuation, the hepatic veins*, that is to say, the vessels which receive most directly the products of digestion, are, after the portal vein, a good deal richer in extractive matters (albuminose) than the rest of the blood; it will be remarked that they contain also more glycose. In nutriments (albuminose, glycose) the richness of the vessels of the liver can be explained by gastric intestinal absorption, to which may be added the prolonged intra-venous digestion, without any agency of the liver whatsoever in the matter."

The author draws certain *pathological deductions* from the preceding physiological laws. To me, his reasoning is at least fair, if not conclusive, and is co-relative with the premises. In connection with albuminoid aliments there exists almost certainly a duodenal indigestion caused by the viciation, insufficiency, or absence of pancreatic secretion; the symptoms of which do not manifest themselves before the end of the second, or commencement of the third hour of digestion. This form of indigestion is accompanied with more profound sensations of inconvenience than gastric dyspepsia. In these cases the internal use of pancreatine is indicated.

The following are some of the secondary causes of duodenal dyspepsia :—

1st. Almost entire insufficiency of that division which the gastric juice effects *at least* in those articles of nourishment that it has not as yet transformed into peptones. The pancreatic digestion is in consequence slower, as gastric oftentimes is, when the teeth have not performed their function. This form of secondary pancreatic dyspepsia is cured by the appropriate treatment for primitive gastric dyspepsia.

"2d. Superabundance of the gastric juice, or insufficiency of the pylorous.

"3d. Insufficiency in the biliary secretion.

"4th. A dyspepsia that may be called portal, or hepatic, resulting from a viciation of intra-venous digestion."

Certain symptoms of dyspepsia, of gastralgia, and hepatalgia, might erroneously be attributed to the stomach, intestine, or liver; but rarely result from the absorption of the pancreatic juice by the portal vein, the secretion being too abundant, active, and irritating.

"When it becomes more urgent to relieve the pain and disturbed functional actions of the digestive organs, than to reëstablish the muscular tone, articles of food which are the most *completely* and *rapidly* dissolved should be preferred, without considering the quantity of peptone that they may furnish.

"When it becomes more desirable to reëstablish the muscular tone than to alleviate the gastro-intestinal troubles, we must then, on the contrary, select a nourishment which, for an equal digestive force, furnishes the greatest amount of peptone, so that it may be more slowly dissolved and digested."

It is useless to give but one kind of azotized aliment for any length of time, not only on account of one kind of nitrogenized nutriment (or peptone) being insufficient to repair the various organs, but also because the same nutriment given exclusively and repeatedly (eight consecutive days, for example) ceases sufficiently to excite the gastric secretions, and no longer entirely undergoes digestive transformation.

The author attributes some of the mistaken notions we have entertained of the pancreatic functions, to the fact of conclusions being arrived at by experimental processes, which are incorrect. For the experiments made by himself, and the unsatisfactory ones of other physiologists, I must refer to the author's text, as they are too numerous to be cited. In speaking of the independent action of the pancreas, he states that the power and influence of the last named organ is such, that in acting upon the nitrogenized elements to transform them into peptones, it can furnish a sufficient quantity of the latter to renovate the entire weight of the body (estimate of the weight being dry) in three hundred days; whilst, on the other hand, the peptones produced by gastric action would accomplish the same result in two hundred days. In order that all may try these experiments, I will give some of the essential conditions laid down by the author as requisite to ensure their success and accuracy.

Supposing we desire to obtain the pancreatic juice by the ordinary method of producing a fistulous opening, and inserting a silver canula into the duct, the following rules must be observed:—

1st. Remembering the *greater rapidity* of pancreatic digestion compared with gastric digestion, we must not prolong pancreatic action beyond four or five hours; otherwise, the conditions will become anti-physiological, because long before this time has elapsed, food submitted inside the body to the action of this juice is not only digested, but has even been absorbed some length of time. Hence, not observing this precaution, putrefaction will necessarily ensue and lead us astray.

2d. To obtain the true and genuine pancreatic juice, capable of digesting, it must not be withdrawn during intestinal fasting. The secretion ceases when the food commences passing into the intestine from the stomach through the pylorus (that is towards the fourth hour after a repast), and reappears when the stomach is empty (which is about the eighth hour).

3d. If we attempt to obtain the experimental results alluded to by an *infusion* from the gland, we must, to be successful, extirpate the gland between the fourth and seventh hours of a good gastric digestion; because if we take it before the fourth hour, its ferment will yet be imperfect; and after the eighth the gland will be exhausted of its contents in consequence of the quantity of pancreatic secretion already furnished. A juice will be obtained, it is true, but not an efficient digestive fluid.

4th. If the juice required to furnish the demonstration should be obtained through the process of a fistula, it is obvious, for the reasons above alluded to, that the fourth hour after a meal should be chosen to perform the operation; otherwise, the stomach, on the one hand, disturbed and irritated, and not being able to accomplish fully its digestive action, will retain the food, and cause the intestine to remain in a state of fasting; on the other hand, the pancreas itself, irritated before the elaboration of its active ferment, will only furnish a vitiated and weak secretion.

In conclusion, it must be remembered that the juice which escapes during the first three hours after the operation is alone to be experimented with. In alluding to the emulsifying property of the pancreas, the author states that those who have *only* succeeded in verifying *this* function have reasoned falsely; because it is impossible to argue against the digestive properties of the pancreatic fluid on nitrogenized food, simply because the pancreas has other functions, such, for instance, as the converting of starchy substances into glucose, or the emulsifying of fats. These properties are entirely distinct. The process of emulsion can go on during *intestinal abstinence*, and is so far from being a *true standard* of pancreatic function, that it can be more readily and largely accomplished *the more vitiated and alkaline the pancreatic juice becomes.*

The pancreas exercises a supplementary function in digesting nourishment which the stomach allows to escape without its having been digested. The pancreas is certainly endowed with a digestive property analagous to that of the stomach, possessing an independent action, and by its power is of equal rank. The author relates an instance, which he witnessed, of a pancreas taken from a man who died suddenly, which digested in four hours almost 3.420 grains of concrete albumen, and 7.752 *grains of fibrine in one hour;* a weight which is equivalent to almost a half day's ration of nitrogenized nourishment allowed to a French cavalry soldier. This, upon a rough calculation, would equal seven ounces and a fraction of albumen, and $1 \frac{249}{720}$ of a pound of fibrine. A very necessary condition for the formation of the pancreatic ferment in abundance, seems to depend upon the *quality* of the blood, which is determined by the presence in the latter of peptones made and absorbed by the stomach.

We are now obliged to conclude this brief review, without noting many points of interest to which we had desired to draw attention. The views of the author have, however, been presented in a condensed form, and if all his experiments have not been described, it must be attributed to want of space and time.

A great part of the subsequent portion of the work is devoted to the description of experiments performed with the principal azotized substances, viz:—albumen, fibrine, caseine, &c. An admirable system has been followed in giving these results, which avoids all confusion. He first takes, for instance, albumen, and subjects it to the influence of the gastric secretion, carefully noting the results; then, entirely reversing the experiment, he places the nitrogenized aliment in the pancreatic fluid, and gives a faithful account of the rapidity and peculiarities of the respective digestions. The last part answers objections made to his experiments and reasoning by those who at first rejected them as inconclusive or erroneous. At some future time we may give additional abstracts, and also the result of some experiments about to be repeated.

